# Unlocking the origins and biology of domestic animals using ancient DNA and paleogenomics

**DOI:** 10.1186/s12915-019-0724-7

**Published:** 2019-12-02

**Authors:** Gillian P. McHugo, Michael J. Dover, David E. MacHugh

**Affiliations:** 1 Animal Genomics Laboratory, UCD School of Agriculture and Food Science, University College Dublin, Dublin, D04 V1W8 Ireland; 2 UCD Conway Institute of Biomolecular and Biomedical Research, University College Dublin, Dublin, D04 V1W8 Ireland

## Abstract

Animal domestication has fascinated biologists since Charles Darwin first drew the parallel between evolution via natural selection and human-mediated breeding of livestock and companion animals. In this review we show how studies of ancient DNA from domestic animals and their wild progenitors and congeners have shed new light on the genetic origins of domesticates, and on the process of domestication itself. High-resolution paleogenomic data sets now provide unprecedented opportunities to explore the development of animal agriculture across the world. In addition, functional population genomics studies of domestic and wild animals can deliver comparative information useful for understanding recent human evolution.

## The origins and evolution of domestic animals

Plant and animal domestication are justifiably considered to be major human cultural innovations that rank in importance with the manufacture of tools, the conquest of fire or the evolution of verbal language. V. Gordon Childe, one of the twentieth century’s greatest archaeologists, considered domestication to be “…that revolution whereby man ceased to be purely parasitic and, with the adoption of agriculture and stock-raising, became a creator emancipated from the whims of his environment” [1].

*Homo sapiens* is not alone in subverting the biology of another species through a process of domestication; leafcutter ant species maintain fungus “gardens” as a source of food [2], while other ant species exploit aphids in a semi-symbiotic interaction in which the ant colony gains honeydew and the aphids gain protection from other insect predators [3]. However, domestication of plants or animals by ancient human populations is categorically different from ant–fungus or ant–aphid mutualisms because it required intentionality and conscious planning and understanding of the behavior and reproductive biology of another species [4, 5]. Therefore, the cognitive demands of human-directed domestication constitute a phenomenon distinct from the interspecific mutualisms evolved by social insects [6, 7].

Wholesale domestication of plants and animals by humans, which began with the wolf (*Canis lupus*) at least 15 thousand years ago (kya) [8–11], was likely triggered by significant environmental and climatic change that accompanied the global transition from the Last Glacial Maximum (LGM) peak approximately 21 kya to the current Holocene interglacial period [12]. It has been proposed that intense climatic variability in the Late Pleistocene would have made food production extremely difficult, if not impossible [13–15]. Conversely, it has been hypothesized that, in the long run, plant and animal agriculture became “compulsory” in the relatively favorable climatic conditions of the Holocene [16], which commenced abruptly at 11.7 kya subsequent to the Younger Dryas, a short 1.2 kya pulse of marked cold and aridity [17]. Intensifying agricultural subsistence strategies during this period exerted pressure on smaller populations that retained less intensive foraging strategies. This generated a competitive ratchet that encouraged the spread of plant and animal agriculture [16]. The demographic pressure of increasing human populations has also been proposed as a causal factor for domestication, resulting in the gradual intensification of relationships between humans and animals over time and culminating in the substantial biological modifications observed in domesticates [6, 18].

**Fig. 1:**
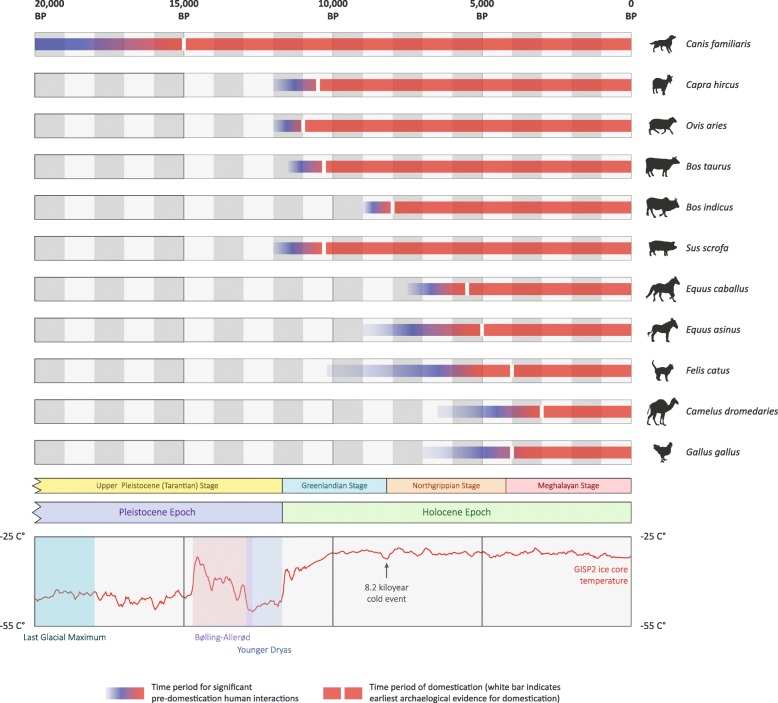
Timelines of domestication for 11 animal species with relevant stratigraphy and climate chronologies. For each species, the time periods of significant pre-domestication human–animal interactions are also shown. Domestication timeline data [4, 5]. Stratigraphy information was obtained from the International Commission on Stratigraphy website [264, 265]. The Quaternary temperature plot was generated from the GISP2 ice core temperature and accumulation data [266–268]

The appearance of the domestic dog (*Canis familiaris*) in the archaeological record was followed relatively soon afterwards by crop and livestock domestication, which allowed humans to substantially augment the food they obtained from hunting and gathering. Consequently, during the Neolithic Transition—the archaeologically documented shift from hunter-gatherer modes of food production to plant cultivation and animal husbandry—increasingly sophisticated agricultural societies developed in multiple locations across Eurasia, North Africa and South and Central America [19, 20]. The zooarchaeology of Southwest Asia indicates that sheep (*Ovis aries*), goats (*Capra hircus*), humpless taurine cattle (*Bos taurus*) and pigs (*Sus scrofa*) were some of the first livestock to be domesticated, 10–11 kya in the Fertile Crescent region [4, 21, 22]. Approximately two millennia later, humped zebu cattle (*Bos indicus*) were domesticated, likely by the early Neolithic cultures located in present-day Baluchistan, Pakistan [4, 23]. Pigs were also separately domesticated about 8 kya in East Asia from a population of wild boar genetically distinct from those in Southwest Asia [4, 24]. The horse (*Equus caballus*) was domesticated on the Central Asian steppes approximately 5.5 kya [4, 25, 26], and the chicken (*Gallus gallus*) and cat (*Felis catus*) went down the same path about 4 kya in Southeast Asia and North Africa (Egypt), respectively [4, 27]. Domestication timelines are shown in Fig. 1 for a range of animal species with corresponding information on key climatic events during the last 20,000 years that likely influenced the emergence of agriculture.

**Fig. 2:**
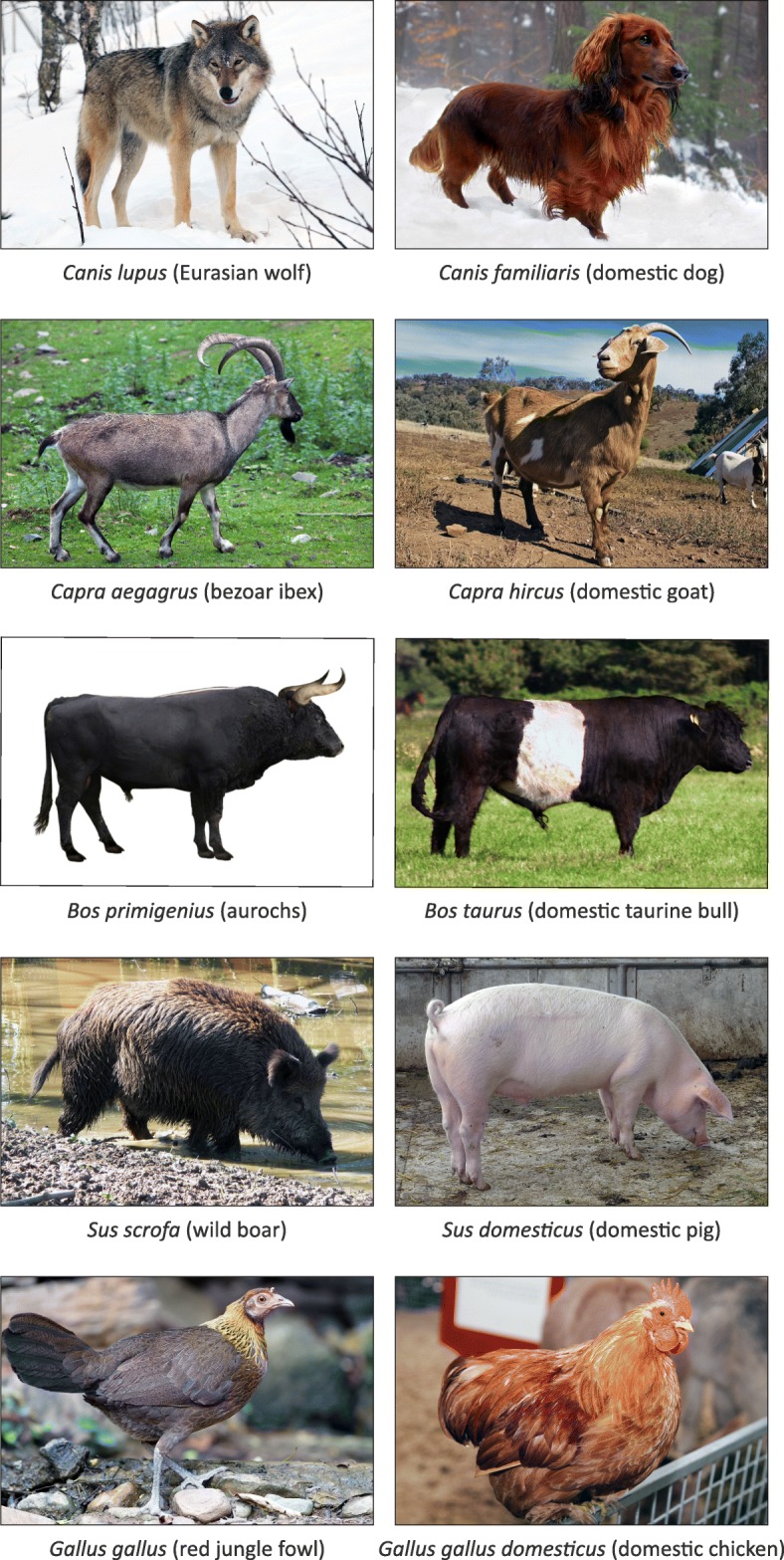
Evolution and phenotypic diversity of domestic animals. The wild progenitor species are shown on the *left* and the domesticated animals are shown on the *right*. Except for the aurochs, all wild progenitor species are extant. The aurochs image is an artistic reconstruction of *Bos primigenius*. Image permissions: wolf (Creative Commons CC BY-SA 4.0); dog (CC BY-SA 2.5); bezoar (ID 79845213©Wrangel | Dreamstime.com); goat (CC BY-NC 2.0 - Fir0002/Flagstaffotos); aurochs and taurine bull (CC BY-SA 3.0); wild boar (CC BY-SA 3.0); pig (public domain), red jungle fowl (CC BY-SA 3.0); and chicken (CC BY-NC 2.0 - Fir0002/Flagstaffotos)

As a well-travelled ship’s naturalist in the 1830s, Charles Darwin enthusiastically observed, studied and catalogued more exotic flora and fauna than almost anyone else living at the time. However, he found the relatively mundane domestic animals of his native island equally fascinating. Darwin’s “long argument” for evolution via natural selection in *On the Origin of Species* was critically underpinned by the analogy between artificial selection of domestic breeds and natural selection in wild populations [28]. Indeed, it has been emphasized that Darwin “found his ‘laboratory’ in the fields and stalls of England” [29, 30]. Stephen Jay Gould, one of the most prolific essayists on Darwinian evolution and biogeography, has noted that the Galápagos finches (*Geospiza* spp.) were not actually discussed in *On the Origin of Species*, and that “…the ornithological star of that great book is the domesticated pigeon” [31]. During the last three decades, studies encompassing molecular population genetics, ancient DNA (aDNA), population genomics and, more recently, paleogenomics, have provided strong support for Darwin’s contention that domestic animal populations and domestication from wild progenitors represent fantastic models for understanding evolutionary processes at a broader level and over longer timescales [32–37]. Fig. 2 shows evolution and phenotypic diversity of domestic animals and corresponding wild ancestral or congener species.

The primary goal of this review is to demonstrate how paleogenomics is revolutionizing our understanding of the origins and biology of domestic animals, including both livestock and companion animals. During the last 10 years there has been an explosion of interest in domestic animal aDNA as sequencing technologies suitable for paleogenomics have become increasingly powerful. We therefore focus on several studies that illustrate the relatively long history of aDNA research in livestock and companion animals. We also use notable published examples to show how paleogenomics is shedding new light on the phylogeography of domestic animals and improving our understanding of the physiological and neurobiological effects of domestication and microevolution of functional traits. In addition, we demonstrate that understanding the genetic origins and spread of domestic animals through analysis of aDNA and paleogenomes can provide new insights into human history, migration and trade. Finally, we propose that paleogenomics and population genomics studies of domestic animals can provide valuable comparative information concerning the paleogenomics and evolutionary history of anatomically modern humans (*H. sapiens*), particularly their interactions with related hominins such as Neanderthals (*Homo neanderthalensis*) and Denisovans (*Homo denisova*).

## Ancient DNA: the beginnings and early studies in domestic animals and related species

Scientists have long speculated about systematically analyzing ancient biomolecules, particularly information-rich molecules such as DNA and proteins (for an early review see [38]). This became technically feasible for aDNA in the early 1980s, albeit through cumbersome molecular cloning methods [39, 40], which ultimately proved unreliable—notably generating spurious DNA sequences from a 2400-year-old Egyptian mummy [40]. A significant breakthrough in the late 1980s was amplification of aDNA from archaeological material and museum specimens using the polymerase chain reaction (PCR) technique, which had recently been developed [41–44]. However, it was also during this time that the significant challenges associated with retrieval of reliable and reproducible aDNA data first began to be appreciated [38, 41, 43, 45]. Consequently, almost from the very beginning, the aDNA field has been beset with significant methodological obstacles including post-mortem damage to preserved biomolecules, contamination of samples and reagents by modern DNA and the presence of inhibitors of enzymatic reactions; all factors that can irrevocably comprise the authenticity and reproducibility of aDNA amplified from archaeological samples [38, 45–50]. However, over the last four decades, as the field of archaeogenetics has matured, scientists have systematically addressed the technical challenges associated with retrieving aDNA from long-dead organisms and it is now well established that vertebrate subfossils can yield authentic and reproducible endogenous molecular genetic information.

The aDNA field has had a long-standing interest in understanding the evolution and biology of domestic animals and their wild relatives [51–55]. The subject of the very first aDNA study published 35 years ago [39] was the quagga (*Equus quagga quagga*), an African equid related to the domestic horse (*Equus caballus*) that was hunted to extinction by the end of the nineteenth century. This work was performed in the pre-PCR era and involved molecular cloning of DNA fragments from dried tissue attached to a quagga skin from a German museum collection. Bacterial colonies containing λ phage vector inserts with mitochondrial DNA (mtDNA) were identified using a mtDNA probe from the mountain zebra (*Equus zebra*) [39]. Following this, two short 117 bp and 112 bp quagga mtDNA clones were sequenced and placed in a phylogeny with mtDNA data from other mammals, thereby opening a whole new scientific discipline of evolutionary archaeogenetics.

The embryonic aDNA field languished as an intellectual curiosity for much of the 1980s; however, this changed rapidly with the introduction of the PCR amplification technique, which kickstarted aDNA research, particularly through work driven by Svante Pääbo and published across a series of seminal papers in 1988 and 1989 [41–44]. Roughly at the same time, parallel work at the University of Oxford by Erika Hagelberg and colleagues showed that aDNA could be retrieved, amplified and analyzed from hard tissues such as bone, which would prove a major boon to the emerging field of molecular archaeology [56–58]. A critical aspect of this work involved retrieval of mtDNA sequences from a 445-year-old domestic pig bone to verify that endogenous DNA could be amplified from hard tissues [57]. In 1990, a French group showed that DNA could also be extracted and analyzed from mammalian teeth [59], again an important technological breakthrough for archaeogenetics in domestic animals.

In common with studies on humans and other vertebrates, the first ancient DNA studies of domestic animals and related species in the 1990s and early 2000s were focused almost exclusively on mtDNA, particularly the hypervariable displacement loop (D-loop) or control region (CR) sequence [51–55, 60–64]. In many respects, mtDNA was ideally suited for the early “proof-of-principle” aDNA-based evolutionary studies: there are hundreds or even thousands of copies in a single animal cell [65] and mtDNA has a markedly higher mutation rate than the nuclear genome [66–68]. It is important to keep in mind that mtDNA represents only a single, non-recombining, maternally transmitted locus. However, PCR amplification of mtDNA from domestic animal subfossils and comparative analyses with extant populations led to some landmark papers and important discoveries, examples of which are described below.

In 1996, Jillian Bailey and her colleagues were the first scientists to recover and analyze ancient DNA from an extinct progenitor of a domestic species, when they sequenced mtDNA from aurochs (*Bos primigenius*), wild cattle that ranged across Eurasia during the Pleistocene and early Holocene [51]. A subsequent study in 2001 corroborated these results with additional ancient mtDNA CR sequence data and also posited a scenario where European aurochs did not contribute to the gene pool of domestic cattle [54]—a hypothesis later disproved by the same group using aurochs nuclear DNA sequence data [69]. The first study of ancient DNA in domestic horses, also published in 2001, used comparative analyses of modern equine mtDNA CR data with sequences from pre-domestic permafrost specimens and Viking-era bones to show extensive retention of diverse ancestral matrilines [55]. These results led the authors to propose a model where domestication was an ongoing process from the late Chalcolithic period through the Bronze Age as the technology for capturing, taming and rearing wild-caught horses disseminated across Central Asia.

In 2002, a comprehensive domestic dog aDNA study was published; using South American and Alaskan specimens that predated European contact, Jennifer Leonard and coworkers showed that mtDNA CR sequence analysis supported the hypothesis that ancient American and Eurasian domestic dogs share a common origin from Old World gray wolves (*C. lupus*) [52]. The first chicken aDNA study ignited a firestorm among archaeologists and paleogeneticists [70]; the authors of this work proposed that mtDNA CR sequence from an archaeological site in Chile provided firm evidence for a pre-Columbian Polynesian introduction of domestic chickens (*G. gallus*) to South America. Additional results from a larger survey of chicken aDNA samples provided support for this hypothesis [71]. However, independent analyses of ancient and modern chicken mtDNA CR sequences robustly disputed this conclusion with suggestions of sloppy laboratory techniques and modern contamination [72, 73], leading inevitably to heated scientific correspondence among the main protagonists [74–77].

As was the case with human archaeogenetics, and population genetics in general, the overreliance on uniparental genetic markers such as mtDNA and Y chromosome polymorphisms led to evolutionary inferences and phylogeographic and demographic reconstructions that, in the long term, could be misleading and generally not robust or well-supported [78–80]. These problems became particularly apparent once high-resolution data became available from the autosomal genome in the form of single-nucleotide polymorphisms (SNPs) and ultimately whole-genome sequence (WGS) data [81–87]. The first phase of aDNA research in domestic animals, therefore, will be remembered for providing tantalizing glimpses of what would ultimately be possible; however, dramatic new technological developments would be required to deliver this ambition.

## Technology advances: deep sequencing + dense bones = paleogenomics

It has long been realized that performing archaeogenetics research correctly is extremely difficult [38, 45, 46, 48–50]. However, by the same token, during the last three decades the challenging nature of aDNA research has spurred significant technical innovation and rapid deployment of state-of-the-art genomics and ancillary technologies [46, 50, 88–93]. Undoubtedly, the most important scientific advance was the introduction of high-throughput sequencing (HTS) to archaeogenetics [94–97]. High-throughput sequencing technologies have been commercially available since 2005 [98] and between 2007 and 2019 there has been an almost 100,000-fold reduction in the raw, per-megabase (Mb) cost of DNA sequencing [99]. Currently, the dominant commercial HTS technology is based on massively parallel sequencing-by-synthesis of relatively short DNA segments [100, 101], which is ideally suited to fragmented aDNA molecules extracted from archaeological and museum specimens. In addition, the vast quantities of sequence data generated—literally hundreds of gigabases (Gb) from a single instrument run—can facilitate cost-effective analyses of archaeological specimens containing relatively modest amounts of endogenous aDNA (for technical reviews see [89–93, 102]).

The introduction of HTS and ancillary specialized methods for sample treatment, aDNA extraction, purification and library preparation have represented a genuinely transformative paradigm shift in archaeogenetics. It has ushered in the era of paleogenomics and the capacity to robustly genotype, analyze and integrate SNP data from thousands of genomic locations in purified aDNA from human and animal subfossils [103–113]. In a comparable fashion to human archaeogenetics [84], the first HTS paleogenomics studies of domestic animals or related species were focused on a single or a small number of “golden samples” [10, 69, 109, 114, 115].

One of the first HTS studies directly relevant to domestic animals was a technical tour de force which pushed the time frame for retrieval of aDNA and reconstruction of paleogenomes beyond 500 kya to the early stages of the Middle Pleistocene [109]. In this study, Ludovic Orlando and colleagues were able to generate a 1.12× coverage genome from a horse bone excavated from permafrost at the Thistle Creek site in north-western Canada and dated to approximately 560–780 kya. Using this Middle Pleistocene horse genome in conjunction with another ancient genome from a 43 kya Late Pleistocene horse, and genome sequence data from Przewalski’s horse (*Equus ferus przewalskii*), the donkey (*Equus asinus*) and a range of modern horses, these authors showed that all extant equids shared a common ancestor at least four million years ago (mya), which is twice the previously accepted age for the *Equus* genus. They also showed that the demographic history of the horse has been profoundly impacted by climate history, particularly during warmer periods such as the interval after the LGM (Fig. 1), when population numbers retracted dramatically in the 15 millennia prior to domestication 5.5 kya. Finally, by focusing on genomic regions exhibiting unusual patterns of derived mutations in domestic horses, it was possible to tentatively identify genes that may have been subject to human-mediated selection during and after domestication [109].

The origins of the domestic dog (*C. familiaris*) and the dispersal of dogs across the globe during the Late Pleistocene and Holocene periods have been extremely contentious, particularly as population genetic, archaeogenetic and paleogenomic data sets have accumulated during the last two decades [8, 116, 117]. Again, like the Thistle Creek horse bone, a small number of key subfossil specimens have provided critical paleogenomic evidence concerning the evolutionary origins of domestic dogs and their genetic relationships with Late Pleistocene Eurasian wolf populations [10, 11, 115]. Pontus Skoglund and colleagues were able to generate a low coverage (~ 1×) nuclear genome from a 35 kya wolf (*C. lupis*) from the Taimyr Peninsula in northern Siberia [115]. Analysis of this Taimyr specimen with WGS data from modern canids showed that this ancient wolf belonged to a population that was genetically close to the ancestor of modern gray wolves and dogs. The results supported a scenario whereby the ancestors of domestic dogs diverged from wolves by 27 kya, with domestication happening at some point subsequent to that event. In addition, this study provided compelling evidence that high-latitude dog breeds such as the Siberian Husky trace some of their ancestry back to the extinct wolf population represented by the Taimyr animal [115].

Another important paleogenome study, published one year after the Taimyr wolf paper, described a high coverage (~ 28×) nuclear genome from a late Neolithic (4.8 kya) domestic dog specimen from Newgrange, a monumental passage grave tomb in eastern Ireland [10]. Analyses of the ancient Newgrange dog genome, additional mtDNA genomes from ancient European dogs and modern wolf and dog genome-wide SNP data suggested that dogs were domesticated independently in the Late Pleistocene from distinct East and West Eurasian wolf populations and that East Eurasian dogs, migrating alongside humans at some time between 6.4 and 14 kya, partially replaced indigenous European dogs [10]. In 2017, following publication of the Newgrange dog genome, Laura Botigué and colleagues generated two ~ 9× coverage domestic dog nuclear genomes from Early (Herxheim, ~ 7 kya) and Late (Cherry Tree Cave, ~ 4.7 kya) Neolithic sites in present-day Germany [11]. Comparison of these two ancient dog genomes with almost 100 modern canid whole genomes and a large genome-wide SNP data set of modern dogs and wolves did not support the dual domestication hypothesis proposed by Frantz et al. one year earlier [10], or the suggested East Eurasian partial replacement of Late Paleolithic or Early Neolithic European dogs.

The origins and fate of the domestic dog populations of the Americas prior to contact with European and African peoples has been the subject of a recent paleogenomics study involving comparisons of ancient and modern dogs. Máire Ní Leathlobhair and colleagues sequenced 71 mitochondrial and seven nuclear genomes from ancient North American and Siberian dogs [118]. Comparative population genomics analyses of these data demonstrated that the first American domestic dogs did not trace their ancestry to American wolves. Instead, however, these pre-contact American dogs (PCDs) represent a distinct lineage that migrated from northeast Asia across the Beringian Steppe with humans more than 10 kya [118]. These analyses also demonstrated that PCD populations were almost completely replaced by European dogs due to large-scale colonization of North and South America within the last 500 years. In a similar fashion to the post-contact human demographic transition in the Americas [119, 120], the authors hypothesize that infectious disease likely played a major role in the replacement of PCDs by European dogs. Finally, they also show that the genome of the canine transmissible venereal tumor (CTVT) cancer lineage, which has evolved to become an obligate conspecific asexual parasite [121], is the closest genomic relative of the first American dogs.

As has been previously noted, understanding the origins and early domestic history of dogs has been complicated by population bottlenecks, expansions, local extinctions and replacements and geographically localized gene flow among wolves and dogs and genetically distinct dog populations [8]. It will, therefore, require systematic large-scale retrieval and analysis of ancient wolf and dog genomes across space and time to accurately reconstruct the evolutionary history of the first animal domesticate [122]. However, this and similar undertakings for other domestic species will be greatly facilitated by another recent technical breakthrough that is described below.

**Fig. 3:**
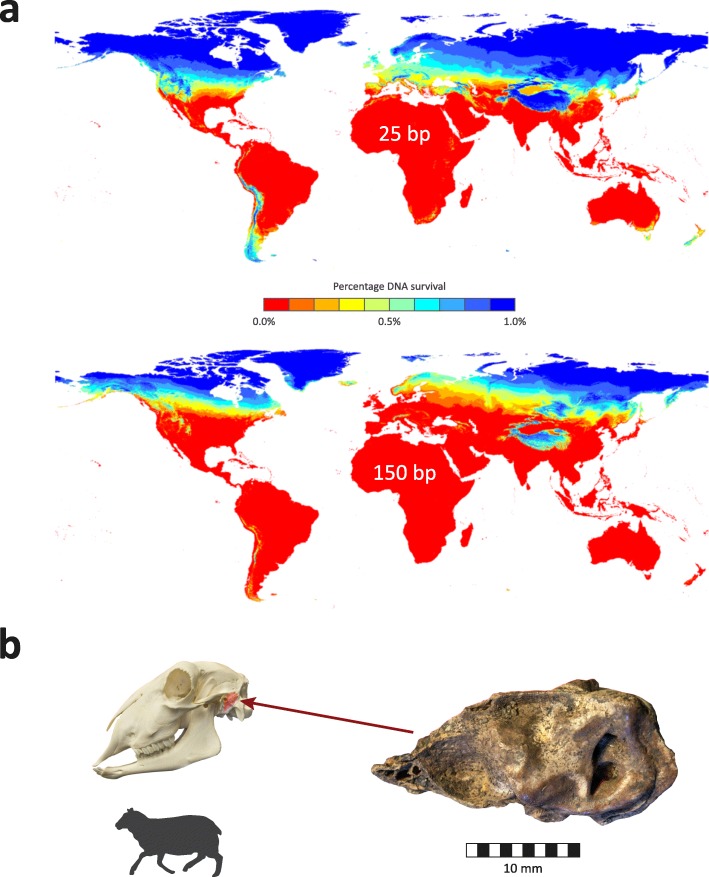
Geography of archaeological DNA survival prior to the discovery of high endogenous DNA content in the mammalian petrous bone. **a** Expected DNA survival after 10,000 years for 25-bp fragments and 150-bp fragments close to the ground surface (modified with permission from [90]). **b** Illustration of a sheep (*Ovis aries*) petrous bone retrieved from a Middle Neolithic site at Le Peuilh, France (modified with permission from [269])

In 2014, a team of Irish geneticists and archaeologists showed that the petrous portion of the temporal bone—the densest bone in the mammalian skeleton—produced the highest yields of endogenous DNA; in some cases, up to 183-fold higher than other skeletal elements [123]. The impact of this discovery has been such that the ancient DNA community now dub the period prior to 2014 “BP” (“before petrous”) [124]. During the last 5 years, DNA extraction from petrous bones, coupled with constantly improving HTS and ancillary technologies, has led to a dramatic scale-up of human archaeogenetics, the cutting edge of which is now the statistically rigorous field of high-resolution population paleogenomics [82, 125–129]. Another notable outcome has been a substantial increase in the proportion of the Earth’s surface area where archaeological excavation can uncover suitable material for successful aDNA extraction and paleogenomics analysis. Previously, for the most part, aDNA research has been confined to regions of the globe where climate and topography were conducive to taphonomic preservation of skeletal DNA (Fig. 3) [90, 130]. However, in recent years human paleogenomics studies have been successfully conducted using samples from arid, subtropical and even tropical zones [131–142].

## Expanding the canvas: population paleogenomics in domestic animals

**Fig. 4:**
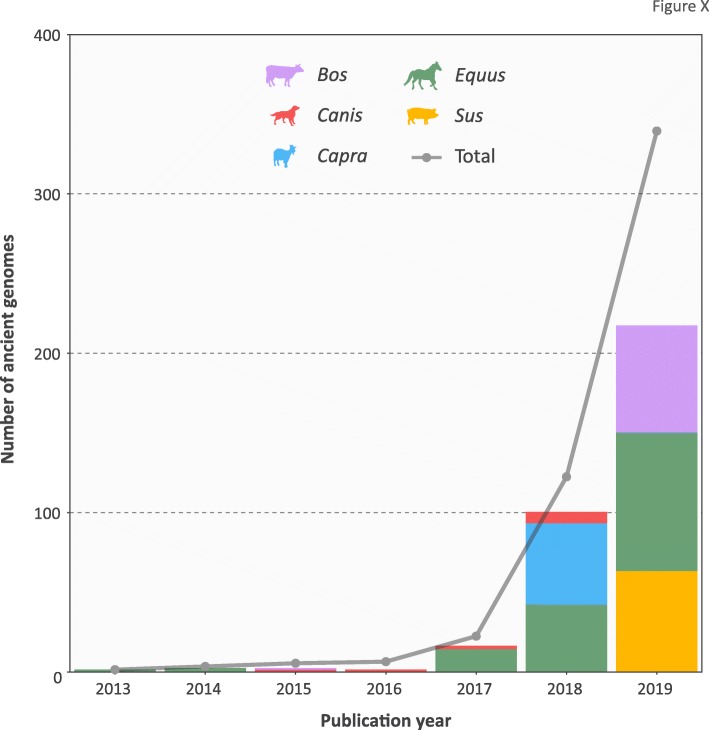
Stacked bar chart and line graph showing the number of ancient samples with whole-genome sequence data (paleogenomes) from domesticated species and their wild relatives. Each genus is represented by a different color and the *line* indicates the total number of paleogenomes generated. The graph was produced in R using ggplot2 (data from [10, 11, 69, 109, 114, 115, 118, 143–146, 169, 191])

Domestic animal paleogenomics has generally followed in the wake of human archaeogenetics and during the last 2 years the first large-scale population-level surveys of ancient livestock genomes have begun to appear [143–146]. This has led to a marked increase in the number of sequenced paleogenomes from domestic animals and their progenitors and congeners (Fig. 4).

Kevin Daly and colleagues were able to generate genome-wide sequence data from four pre-domestic goats (bezoars—*Capra aegagrus*) and 47 domestic goats (*C. hircus*) excavated from sites traversing Southeastern Europe and the Near East and spanning almost 50,000 years from the mid-Upper Paleolithic (> 47 kya) to the early modern period (~ 0.5 kya). It is also notable that many of these goat subfossils were petrous bones excavated from archaeological sites where summer temperatures regularly exceed 35 °C. The diversity of bezoar and goat mtDNA and nuclear genomes across Southeastern Europe and the Near East supports the hypothesis that goat domestication in the Near East took place over an extended period of time and in a spatially dispersed manner, which is contrary to a simplified Vavilovian model of a single core domestication zone with radial dispersal of early domesticates. These observations mirror paleogenomics data from early Neolithic farmers, which also show discontinuous genomic diversity across the region [133, 147–149].

From a functional population genomics perspective, detection of outlier genomic loci exhibiting signatures of selective sweeps identified several plausible candidate genes that may have undergone rapid microevolution during and soon after goat domestication. Prominent among these were genes for pigmentation proteins such as the KIT proto-oncogene receptor tyrosine kinase (encoded by *KIT*) and KIT ligand (encoded by *KITLG*). Early human-mediated selection at these loci may have been to facilitate visual recognition of individual animals, or as a pleiotropic consequence of breeding for behavioral traits such as tameness (see the following section). In addition to pigmentation and other signals associated with growth and reproduction, Daly and colleagues identified an intriguing selection signature centered on the caprine ortholog of the human cytochrome P450, family 2, subfamily C, polypeptide 19 gene (*CYP2C19*), which has been implicated in metabolism of a mycotoxin produced by *Fusarium* spp. that cause Fusarium ear blight disease in cereals. They hypothesized, therefore, that a caprine *CYP2C19* variant that protects against this toxin would have been under positive selection in response to a diet containing increasing amounts of cereal waste byproducts [143].

Additional high-resolution population-level studies of domestic and wild paleogenomes have recently been published that illustrate the power of this approach in providing new insight on the origins, biogeography and functional biology of mammalian livestock [144–146]. For example, Antoine Fages and colleagues analyzed a very large genome-wide sequence data set generated from 278 domestic equid subfossils that span the last 6000 years [144]. A notable outcome from this work is strong support for the hypothesis that the advent of agricultural mechanization and motorized transport led to a marked decrease in genomic diversity of modern horses compared to populations that existed prior to the Industrial Revolution. Examining patterns of genomic variation further back in time also revealed that the influence of Persian-derived lineages increased following the expansions of Islamic cultures in the second half of the first millennium CE. In addition, evaluation of positive selection using population branch statistics showed that by the second millennium CE there was evidence for significant changes in genes regulating skeletal development and anatomy. Finally, this study uncovered two additional horse lineages that existed during the fifth millennium BCE at the northeastern and southwestern extremities of Eurasia, but which became extinct with minimal genetic contributions to modern domestic horses.

A similarly in-depth study of domestic and wild paleogenomes but with a geographical focus on the Fertile Crescent and surrounding regions has also shed new light on the domestic origins and spread of cattle during the Neolithic period and in subsequent millennia [146]. Using WGS data from 67 ancient cattle, including six aurochs (*B. primigenius*) specimens and genome-wide SNP data from modern cattle, Marta Verdugo and her colleagues were able to investigate the domestic history and microevolution of cattle across nine millennia beginning at the latter stage of the seventh millennium BCE. They showed that there was significant male-mediated gene flow from arid-adapted zebu cattle (*B. indicus*) into the greater Near East, which commenced with a multi-century period of drought that began 4.2 kya and marks the beginning of the recently ratified Meghalayan stage of the Holocene Epoch [150]. In addition, analyses of WGS data from ancient domestic cattle that inhabited the southern Levant and a Moroccan aurochs specimen dated to approximately 9 kya demonstrated that a distinct subpopulation of aurochs ranged across the Levant and the North African littoral. This led the authors to hypothesize that the previously recognized genetic distinctiveness of African *B. taurus* cattle [54, 151, 152] may stem from roots in the southern Fertile Crescent.

Laurent Frantz and his colleagues have recently published the first comprehensive population paleogenomics study of wild and domestic pigs in the Near East and Europe [145]. Using 63 nuclear paleogenome data sets in conjunction with mtDNA sequences from more than 2000 modern and ancient animals, they were able to reconstruct a detailed genetic history for *S. scrofa* in western Eurasia over the last 14 millennia. The most notable outcome from this work was confirmation that the domestic pig populations that have inhabited mainland Europe for approximately 8 kya have undergone a complete genomic turnover via gene flow from indigenous wild boars that was particularly rapid during the centuries after first contact. This process had been suggested by earlier studies of modern and ancient mtDNA [63] and by medium-density SNP array data from European and Near Eastern wild boar and three European domestic pig populations [153]. However, it required the extensive paleogenomics data generated by Frantz et al. [145] to tease out the chronology and dynamics of admixture between wild boar and the early domestic pig populations of Europe.

The functional impact of wild boar introgression was also assessed through comparative analyses of haplotypes previously reported to have been subject to human-mediated selection [24]. These analyses demonstrated that the small proportion of retained Near Eastern genomic ancestry in modern European pigs has not been specifically targeted by selective breeding. One exception to this general trend, however, may be the D124N variant of the melanocortin 1 receptor protein (encoded by *MC1R*) associated with black (or black and white spotted) pigmentation in many western Eurasian domestic pig breeds. This non-camouflage coat-color phenotype has been maintained in the face of substantial gene flow from wild boar and phylogenetic analyses of the genomic region surrounding *MC1R* led Frantz and colleagues to hypothesize an origin for the D124N variant in Anatolian domestic pigs more than 8 kya [145].

## Interrogating paleogenomes to understand the biology of animal domestication

The vanguard of high-resolution surveys of livestock paleogenomes described in the previous section signpost the future of archaeogenetics in domestic animals. They point towards high-resolution studies across time and space that will reveal the genetic architecture of animal domestication and the physiological and neurobiological changes that occur as livestock and companion animals are brought under human control and subject to long-term reproductive management and artificial selection. It is likely that high-resolution surveys of pre-domestic and early animal paleogenomes will provide important new information on intriguing features of domestic animals and the domestication process that were first highlighted by Charles Darwin more than 150 years ago [154].

Because of his interest in human-mediated breeding and selection, Darwin had spent many years studying behavioral, physiological and morphological traits in domestic animals. He observed that the diverse range of domesticated mammals—rodents, lagomorphs, carnivores, artiodactyls and perissodactyls—exhibit a shared collection of developmental, anatomical, physiological and behavioral traits that set them apart from wild mammals. This “domestication syndrome” now encompasses a catalogue of biological features that include pedomorphosis with increased tameness and docility; reduction in sexual dimorphism; modifications to craniofacial morphology and decreased brain size; dramatic coat color variation and depigmentation; non-erect floppy and small ears; and alterations of the endocrine system with significant changes to female reproductive physiology, particularly frequent and nonseasonal estrus cycles [155–157].

As an explanatory framework for a deeper understanding of this phenomenon, it has been hypothesized [158–161] that domestication has selected for pre-existing and novel genomic variants that perturb the gene regulatory networks (GRNs) underpinning ontogeny of the myriad tissues and anatomical structures derived from the vertebrate neural crest stem/progenitor cell population [162–164]. The neural crest hypothesis proposes that traits associated with the domestication syndrome have a shared developmental basis. This is due to the role of stem cells from the crest or dorsal edge of the neural tube of vertebrate embryos, which ultimately form or influence a range of anatomical features, and neurobiological and physiological processes [161]. The neural crest hypothesis has recently been supported by comparative studies of whole-genome sequence and SNP data from domestic dogs, cats and foxes (*Vulpes vulpes*) and their wild counterparts [165–168]. These studies demonstrated that some of the genes in these species that exhibit signatures of selection due to domestication are embedded in the GRNs that determine the fate of neural crest cells during early embryonic development.

To date, only one in-depth paleogenomics study has provided convincing evidence in support of the neural crest hypothesis. Pablo Librado and colleagues examined a series of 14 Central Asian domestic horse paleogenomes spanning the Bronze and Iron Ages between 4.1 and 2.3 kya [169]. They applied a novel statistical method based on *levels of exclusively shared differences* (LSD) for genome-wide selection scans that can identify loci that underwent selection in a population with high sensitivity and specificity [170]. Comparisons of the Bronze and Iron Age horse paleogenomes with groups of pre-domestic, modern domestic and Przewalski’s horse (*E. f. przewalskii*) using the LSD method identified genes positively selected during the early domestication process. Genes detected as enriched by this approach included genes related to ear shape, neural crest cell morphology, neural mesenchyme and neural crest-derived neurons involved with movement, learning and reward [169]. In particular, these analyses highlighted the treacle ribosome biogenesis factor 1 (*TCOF1*), KIT ligand (*KITLG*) and fibroblast growth factor receptor 1 (*FGFR1*) genes associated with neural crest cell development and regulation. In the coming years, it is likely that high-resolution surveys of paleogenomes across time and space in other species will shed further light on the role of neural crest cell GRN perturbation in animal domestication. It should also be possible to determine if this process is universal across mammalian livestock and companion animals, and whether it also extends to other domestic vertebrates such as birds and fish [161].

Based on progress during the past decade, paleogenomics combined with comparative evolutionary genomics will provide a deeper understanding of the genetic architecture, neurobiology and physiology of mammalian domestication [33–37]. In this regard, it has been proposed that treating modern humans as “self-domesticated” could provide a new avenue to understanding both early and recent human evolution [171–173]. Unsurprisingly, the original idea that modern humans are self-domesticated can also be attributed to Charles Darwin; however, he remained equivocal as to whether the unusual biology of our species could really be associated with the same processes that gave rise to domestic animals [174]. In addition, later scientists were generally hostile to the concept; for example, in 1962, Theodosius Dobzhansky wrote “… ‘domestication’ of man is too vague an idea to be scientifically productive” [175]. Recently, however, as the field of domestication studies has advanced, the hypothesis of human self-domestication is increasingly being revisited—particularly with regards to the evolution of prosociality and language [176–181]. The rapid accumulation of paleogenomes from early domestic animals, and anatomically modern humans, Neanderthals and Denisovans, would therefore suggest that the self-domestication hypothesis can finally be rigorously tested and assessed using high-resolution comparative genomics.

Another feature of domestication that has been explored using population genomics and paleogenomics of livestock and companion animal populations is the documented increase in deleterious genetic variation that has been termed the “cost of domestication” [182, 183]. The intellectual roots of this concept can again be traced back to Charles Darwin and also to Alfred Russel Wallace, both of whom suggested that the benign “conditions of life” for domestic animals may ultimately have negative consequences in terms of evolutionary fitness [154, 184]. Prior to the genomics era, theoretical population genetics models predicted domestication and artificial selection would lead to accumulation of deleterious alleles and an increase in the genetic load through genetic hitchhiking [185], population bottlenecks that negatively affect purifying selection [186] and reductions in locus-specific effective population size [187].

In recent years, comparisons of genome sequence data from domestic dogs, yaks (*Bos grunniens*), rabbits (*Oryctolagus cuniculus*) and chickens with their wild congeners have supported the cost of domestication hypothesis [188–190]. Again, equine studies have led the way in investigating the cost of domestication using paleogenomics data. Mikkel Schubert and colleagues compared two ancient pre-domestic Asian horse genomes sequenced to relatively high coverage (7.4× and 24.3×) with modern genomes. They observed significantly increased deleterious mutation loads in the extant genomes that could not simply be attributed to increased rates of inbreeding in present-day horse populations [114]. It is important to note, however, that more extensive tracking of genomic variation across time has shown that the mutational load in modern horses has accumulated relatively recently, presumably because of selective breeding practices that have become increasingly sophisticated over the centuries [144, 169, 191].

Notwithstanding the general pattern observed for other species, European pigs seem to have escaped the genetic load imposed by domestication and artificial selection through long-term gene flow from wild boar and more recent admixture with East Asian pig populations [190]. This leads us to the next important contribution of paleogenomics to understanding the origins and genetic history of domestic animals.

## Multiple melting pots: reticulate gene flow and admixture in domestic animals

The 2010 paper [103] from Svante Pääbo’s group describing the draft Neanderthal nuclear genome—a seminal contribution to our understanding of recent human evolution—was followed swiftly the same year by publication of the Denisovan genome, which was arguably even more revelatory [105]. Comparative analyses of these paleogenomes provided surprising but convincing evidence of reticulate gene flow and admixture between these archaic groups and anatomically modern humans during the Late Pleistocene [103, 105]. Additional Neanderthal and Denisovan genome sequence data have been assembled over the past decade, some of which are at sufficiently high depth for functional population genomics investigations of adaptive and maladaptive introgression into modern human populations (for reviews see [129, 192–196]). It is now well established that people outside of sub-Saharan Africa exhibit varying but consistently detectable genomic signatures of admixture with these archaic hominins [82, 126, 129, 192, 194–196]. In addition, introgression of Neanderthal and Denisovan protein-coding gene segments and genomic regulatory elements (GREs) has had functional consequences, the textbook example being positive selection of a Denisovan haplotype of the endothelial PAS domain protein 1 gene (*EPAS1*) for altitude adaptation in Tibetan human populations [197].

**Fig. 5:**
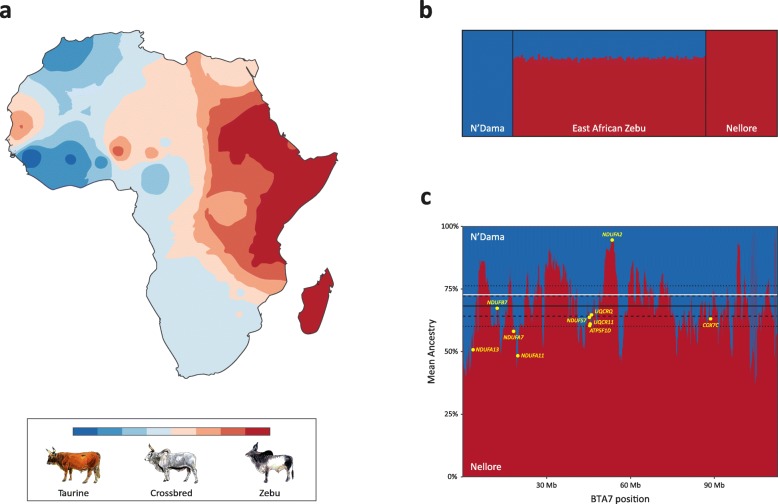
Taurine–zebu admixture and genomic introgression in hybrid African cattle. **a** Interpolated synthetic map illustrating spatial distribution of admixture, which is generated from the first principal component (PC1) of a principal component analysis (PCA) of genetic variation across African cattle populations (modified with permission from [205]). **b** Genetic structure plot generated from high-density SNP data (Illumina BovineHD BeadChip with 777,962 SNPs) showing individual animal proportions assuming two source populations (N’Dama, *n* = 24; East African Zebu, *n* = 92; Nellore, *n* = 34) (the authors, unpublished results). The structure plot was generated using fastSTRUCTURE [270] and visualised using DISTRUCT [271]. **c** Chromosomal local ancestry plot for bovine chromosome 7 (BTA7) showing *Bos taurus* and *Bos indicus* ancestry in East African Zebu cattle (the authors, unpublished results). Nuclear oxidative phosphorylation (OXPHOS) genes are highlighted, illustrating the potential of admixed cattle for evaluating mitonuclear disequilibria. The plot was generated using the efficient local ancestry inference (ELAI) method [272]

In a comparable fashion to studies of modern and archaic humans, high-resolution population genomics and paleogenomics have begun to demonstrate that the evolutionary origins and genetic history of domestic animals are generally more complex and scientifically intriguing than the relatively simplistic scenarios originally posited using small numbers of uniparental genetic markers and autosomal polymorphisms [8, 25, 33–36, 198]. An early and instructive example derives from relatively comprehensive surveys of a single uniparental marker (mtDNA) in extant cattle populations [151, 199, 200]. This work clearly demonstrated the substantial evolutionary divergence between *B. taurus* (taurine) and *B. indicus* (zebu) cattle that had previously been hinted at by protein polymorphism data [201, 202]. However, the early focus on mtDNA meant that detecting and disentangling sex-biased hybridization and extensive zebu–taurine admixture in African and Middle Eastern cattle populations only became possible with the availability of both modern and ancient nuclear genetic marker data [146, 152, 203–205]. Fig. 5 shows patterns of admixture between taurine and zebu cattle in Africa at geographic, population and genomic scales. In this regard, the estimated evolutionary divergence between the *B. taurus* and *B. indicus* lineages, at 0.2–0.5 kya [206–208] suggests that taurine–zebu hybridization and adaptive introgression may ultimately provide a useful genomic framework for understanding admixture and subchromosomal local ancestry in other mammalian species, including archaic hominins and anatomically modern humans. For example, mitonuclear interactions with biochemical and physiological impacts, which can be examined at high resolution in hybrid African cattle (Fig. 5), have also recently been described in admixed modern human populations [209] and for maladaptive Neanderthal haplotypes at human nuclear loci [210].

**Fig. 6:**
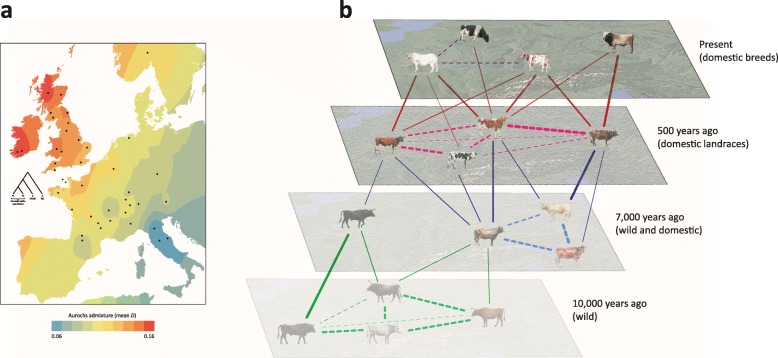
Reticulate evolution in European wild aurochs and domestic cattle. **a** Geographic contour map of localized ancient British aurochs (*Bos primigenius*) genomic admixture with modern European cattle breeds (modified from [69] under the terms of the Creative Commons Attribution 4.0 International License, http://creativecommons.org/licenses/by/4.0). **b** Spatio-temporal model of historical admixture and gene flow in European cattle populations

The problems associated with overreliance on mtDNA sequence diversity data are also encapsulated in one of the first aDNA studies of ancient wild cattle, which concluded that native European aurochs (*B. primigenius*) did not contribute to the gene pool of domestic cattle [54]. However, it was only when WGS data became available from a pre-domestic northern European aurochs that a more nuanced scenario of localized gene flow became apparent [69, 211]. In our opinion, therefore, once paleogenomic data are assembled for wild and early domestic cattle across Eurasia, this pattern will crystallize into a spatio-temporal mosaic of reticulate aurochs admixture and introgression that may have profound consequences for understanding phenotypic diversity in modern cattle populations. Fig. 6 illustrates this model, which may also be applicable to other domestic livestock as they migrated with early agriculturalists and encountered related wild species. For example, the recent work of Verdugo and colleagues described above that encompassed analyses of cattle paleogenomes from the Fertile Crescent and surrounding areas revealed an intricate pattern of admixture and introgression over time [146].

Complex reticulate evolutionary histories have come into focus for other domestic animals and wild congeners during the past decade—over both long and short evolutionary timescales [10, 24, 115, 206, 208, 212–222]. In a remarkable example of convergent adaptive introgression—mirroring ancient genetic exchange between Denisovans and humans—a canine *EPAS1* variant from altitude-adapted gray wolves has been selected in mastiff dogs that have inhabited the Tibetan Plateau for hundreds of years [215, 216].

Domestic pigs, wild boar and other suid species also have a highly complex reticulate and multilayered history of intraspecific and interspecific admixture [223, 224]. In the first instance, genome sequence data have provided convincing evidence for ancient admixture and gene flow over a relatively long timescale among *S. scrofa* and other *Sus* spp. across the islands and mainland of Southeast Asia where the group first evolved more than 4 mya [219, 225]. Secondly, two separately domesticated major west and east Eurasian lineages of domestic pigs share a common ancestor more than 1 mya [221, 225, 226] and have been subject to extensive human-mediated crossbreeding to enhance traits of commercial interest, particularly in northern European production pig breeds [221, 227–231]. Thirdly, since the early Neolithic, the genetic composition of pig populations across Eurasia has been profoundly influenced by recurrent gene flow from wild boar [24, 63, 232–237]. In particular, analyses of aDNA from archaeological material have shown that there was mtDNA turnover with wild boar as early domestic pigs migrated into Europe during the Neolithic [63, 235, 237]. Finally, an additional layer of complexity became evident with detection of back migration and introgression of European mtDNA haplotypes into Bronze and Iron Age Middle Eastern domestic pig populations [236–239]. It is important to note, however, that the complex genetic history and biogeography of domestic pigs during the Holocene will only become understood with detailed spatio-temporal paleogenomics data from across Eurasia and beyond. In this regard, the recent study by Frantz and colleagues described above is an important first step towards this goal [145].

## Forward to the past: the outlook for archaeogenetics in domestic animals

During the past decade progress in archaeogenetics has been driven by spectacular technology developments in genomics and other fields. This has led to the establishment of paleogenomics “factories” for studying recent human evolution, migration and admixture at increasingly high resolution [240]. There have also been significant developments in other areas of biomolecular archaeology, some of which we outline below in the context of understanding the genetic history and recent evolution of domestic animals.

Ancient DNA may also be readily extracted from a wide range of museum specimens containing biological material from domestic animals [241–243]. However, it is important that minimally or non-destructive sampling methods are employed for these items, many of which are literally irreplaceable [244, 245]. Novel sources of aDNA such as avian eggshells and feathers [246], animal glues [247] and parchment made from processed livestock skins [248, 249] will likely have a major impact on archaeogenetics studies of domestic animals. Written documents made from parchment have been carefully maintained and curated for many centuries and therefore represent a valuable repository of genomic information that could illuminate livestock agriculture, breeding and trade stretching back to the early Middle Ages [249].

The expansion of livestock paleogenomics studies to encompass wide spatio-temporal surveys of archaeological material will provide new information concerning the development of secondary animal products and resources such as milk, wool, traction and transport that can be repeatedly exploited throughout an animal’s lifespan [250, 251]. Over the coming years it is likely that high-resolution paleogenomics will shed light on human-mediated selection and the phenotypic changes in livestock that underpinned the “Secondary Products Revolution” in early agricultural societies [252]. Another major area of growth during the coming decade will be identifying and analyzing microbial pathogen genomes using archaeological material from domestic animals and wild congeners [253, 254]. This approach will provide new information for infectious disease research in livestock and companion animals, particularly for diseases such as bovine tuberculosis caused by *Mycobacterium bovis*, which may have emerged as livestock population densities increased during the Neolithic period [255].

The introduction of aDNA and particularly paleogenomics to archaeology has not been universally welcomed [256]. In this regard, some commentators have proposed a “new archaeology”, which suggests that the role of archaeologists in population paleogenomics should be to ensure geneticists are fully informed about the complexities of human actions, interactions and population movements during the past [257]. Accordingly, this multidisciplinary approach would fully encompass existing scholarship on human history and prehistory, thereby facilitating accurate interpretations of paleogenomics data from ancient peoples and their animal companions [258–260]. Going forward, therefore, it will be important to ensure that archaeologists and historians are actively involved in large-scale paleogenomics studies of livestock and other domestic animals, and that these experts are considered to be more than just passive “sample providers” [256, 261].

It is important to finish this review by emphasizing that there will be myriad practical applications for systematically exploring and cataloguing domestic animal genome diversity using high resolution population genomics of extant and extinct domestic animal populations and their wild ancestors. For example, the Functional Annotation of Animal Genomes (FAANG) initiative that aims to identify all functional elements in animal genomes [262] will directly benefit from understanding how genomic regulatory networks have been shaped by domestication, migration and adaptive introgression from wild populations, as well as ancient and more recent human-mediated selection. Finally, identifying and tracking functionally important genomic variation in livestock across space and time will provide novel information for enhancement of welfare, health and production traits using new breeding technologies that are underpinned by genome editing [263].

## Data Availability

Not applicable.
